# Enumeration method for tree-like chemical compounds with benzene rings and naphthalene rings by breadth-first search order

**DOI:** 10.1186/s12859-016-0962-4

**Published:** 2016-03-01

**Authors:** Jira Jindalertudomdee, Morihiro Hayashida, Yang Zhao, Tatsuya Akutsu

**Affiliations:** Bioinformatics Center, Institute for Chemical Research, Kyoto University, Uji, Gokasho Japan

**Keywords:** Benzene ring, Naphthalene ring, Enumeration, Breadth-first search

## Abstract

**Background:**

Drug discovery and design are important research fields in bioinformatics. Enumeration of chemical compounds is essential not only for the purpose, but also for analysis of chemical space and structure elucidation. In our previous study, we developed enumeration methods *BfsSimEnum* and *BfsMulEnum* for tree-like chemical compounds using a tree-structure to represent a chemical compound, which is limited to acyclic chemical compounds only.

**Results:**

In this paper, we extend the methods, and develop *BfsBenNaphEnum* that can enumerate tree-like chemical compounds containing benzene rings and naphthalene rings, which include benzene isomers and naphthalene isomers such as ortho, meta, and para, by treating a benzene ring as an atom with valence six, instead of a ring of six carbon atoms, and treating a naphthalene ring as two benzene rings having a special bond. We compare our method with MOLGEN 5.0, which is a well-known general purpose structure generator, to enumerate chemical structures from a set of chemical formulas in terms of the number of enumerated structures and the computational time. The result suggests that our proposed method can reduce the computational time efficiently.

**Conclusions:**

We propose the enumeration method BfsBenNaphEnum for tree-like chemical compounds containing benzene rings and naphthalene rings as cyclic structures. BfsBenNaphEnum was from 50 times to 5,000,000 times faster than MOLGEN 5.0 for instances with 8 to 14 carbon atoms in our experiments.

## Background

Enumeration of chemical compounds is important in bioinformatics, and has been adapted to several applications such as drug discovery and design [1–3], structure elucidation [4–6], and analyses of chemical spaces [7–13]. It is defined as a problem of generating all non-redundant chemical structures satisfying some constraints. For example, a chemical formula, which consists of the number of each atom included in the compound, is given as an input. There are several algorithms for enumerating chemical compounds from a chemical formula and most of them use a molecular graph to represent a chemical compound, where the nodes and edges of the graph refer to atoms and bonds of the chemical compound, respectively. Some of those algorithms are claimed to be able to enumerate various chemical structures without restriction of the structure, such as MOLGEN [14] and Open Molecule Generator (OMG) [15]. It was reported that OMG is able to deal with different valences for a kind of atom, and was not efficient for several instances compared with MOLGEN. While the remaining ones, such as EnuMol [16, 17] as well as *BfsSimEnum* and *BfsMulEnum* [18], have a limitation of the structure of enumerated compounds, such as acyclic compounds for BfsSimEnum and BfsMulEnum and compounds with no cycle except for benzene rings for EnuMol, the methods consume significantly less computational time. There are also related application softwares, e.g. SmiLib [19] and CLEVER [20], that generate chemical compounds from given fragments. The limitation of these tools is that they require a library of desired chemical fragments, which can be generated by the enumeration tool.

Our previous methods, BfsSimEnum and BfsMulEnum, use a tree structure, instead of a general graph, to represent a chemical compound and call it *a molecular tree* so they can generate only tree-like chemical compounds. In this work, we develop *BfsBenNaphEnum*, which aims to reduce the limitation of previous methods by extending them such that they can enumerate chemical compounds containing only benzene rings and naphthalene rings as cyclic structures, which are six carbon atoms cyclic structures and ten carbon atoms bicyclic structures, respectively. Pólya proposed a group-theoretic method for isomer counting of single cyclic structures such as a benzene ring, a naphthalene ring, and an anthracene ring using the cycle index, from which many studies followed [21]. However, structures enumerated by these methods are restricted to certain types. Indeed, Meringer wrote that up to now the only way to calculate the number of isomers belonging to an arbitrary molecular formula is to use structure generators [22]. Suzuki et al. considered the problem of enumerating structures having monocyclic graph structures, each of which has exactly one cycle [23]. An enumeration method for tree-like chemical compounds containing only benzene rings as cyclic structures has been implemented on EnuMol web server (http://sunflower.kuicr.kyoto-u.ac.jp/tools/enumol/). On the other hand, our method can enumerate compounds containing naphthalene rings in addition to benzene rings. Moreover, the proposed algorithm can calculate the number of benzene rings and naphthalene rings from chemical formula, while users have to specify the number of benzene rings in EnuMol.

Chemical structures considered in this study can be represented by a molecular tree, where a benzene ring is converted to a node with valence six and a naphthalene ring is considered as two benzene nodes having a special bond. We name that special bond as *a merge bond*. Since a merge bond merges two carbon atoms of two benzene rings together, it reduces the number of carbon atoms with free valence electron of two benzene rings by two so we represent a merge bond by a double-edge. Moreover, benzene nodes cannot have double bonds with other nodes because they bond with other non-benzene atoms by a single bond [24]. This means that a double-edge represents a double bond if it connects two non-benzene nodes, while it represents a merge bond if it connects two benzene nodes. Therefore, bonds in a benzene ring and a naphthalene ring are considered as the same bond and Kekulé representation is not included in this work. Besides, this work uses a two-dimensional molecular tree to represent a chemical structure so it cannot deal with stereoisomers. For tautomeric, this work considers two structures in a pair of tautomeric as non- redundant compounds and generates both of them.

BfsSimEnum and BfsMulEnum are modified to return a set of molecular trees as the output, given a chemical formula, the number of benzene rings, and the number of naphthalene rings. After that, an attribute called *carbon position list* is added into benzene nodes in a molecular tree to represent the way that benzene nodes bond with their adjacent nodes. This attribute is important because bonding with different carbon atoms in a benzene ring may result in different chemical structures. Finally, for each molecular tree from BfsSimEnum and BfsMulEnum, we generate a set of molecular trees whose nodes adjacent to benzene nodes are labeled with a carbon position such that all chemical structures are enumerated without redundancy based on normal form rule.

For evaluating our proposed method, we perform computational experiments for several instances, and compare the execution time by our method with that by MOLGEN. We show that our proposed method is efficient for enumerating chemical compounds containing benzene rings and naphthalene rings, and is from 50 times to 5,000,000 times faster than MOLGEN for several instances in our experiments.

## Preliminaries

### Enumeration problem

Let *Σ* be a finite set of labels of atoms, for example, *Σ*={C,N,O,H }, where ‘C’, ‘N’, ‘O’, and ‘H’ denote carbon, nitrogen, oxygen, and hydrogen atoms, respectively. A *molecular graph* is defined as a multi-graph *G*(*V, E*), where *V* is a set of nodes and *E* is a set of multi-edges, also denoted by *V*(*G*) and *E*(*G*), respectively. Each node is labeled with an atom-label in *Σ*, while each edge represents the bond between two atoms and the multiplicity of edge represents the bond type. The degree of each node is equal to the valence of its atom. Let *deg*(*v*) and *l*(*v*) be the degree and the label of node *v*, respectively. Let *val*(*l*_*i*_) be the valence of the atom represented by label *l*_*i*_ in *Σ*. It should be noted that there exist different valences for a kind of atom, for example, carbon atoms of CO_2_ and CO. For this case, it is sufficient to put two distinct labels *C* and *C*^(2)^ in *Σ*, and to define *val*(*C*)=4 and *val*(*C*^(2)^)=2. Let *num*(*G,l*_*i*_) be the total number of nodes labeled with label *l*_*i*_ in molecular graph *G*. Then, the enumeration problem is defined as follows.

#### **Problem1**.

Given the numbers $n_{l_{i}}$ of atoms for all labels *l*_*i*_∈*Σ*, the number *n*_*b*_ of benzene rings, and the number *n*_*n*_ of naphthalene rings, enumerate all non-redundant molecular graphs *G* such that $num({G,l}_{i}) = n_{l_{i}}\phantom {\dot {i}\!}$ for all *l*_*i*_∈*Σ*, *deg*(*v*)=*val*(*l*(*v*)) for all nodes *v*∈*V*(*G*), and *G* includes exactly *n*_*b*_ benzene rings, *n*_*n*_ naphthalene rings, and no other cyclic structures. It must be noted that *n*_*b*_ and *n*_*n*_ can be zero.

In the case that the input chemical formula contains five or less carbon atoms, BfsStructEnum can enumerate only tree-like chemical compounds by specifying the number of benzene rings and the number of naphthalene rings to be zero. Because we enumerate molecular trees such that degree of each node equals to valence of atom label of that node, charged molecules cannot be enumerated automatically. However, they can still be enumerated by specifying a charged atom as a new kind of atom type with appropriate valence value.

**Fig. 1:**
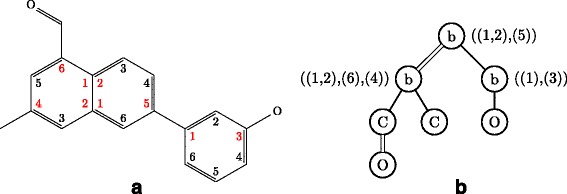
Example of a molecular graph including benzene rings and naphthalene rings. **a** A molecular graph including one benzene ring and one naphthalene ring. **b** A rooted tree contracted from the left graph. It is noted that hydrogen atoms are omitted

Since our enumeration methods deal with a chemical compound as a node-labeled rooted ordered tree for efficient enumeration, we contract cyclic structures appearing in a molecular graph to single nodes. Concretely, we contract a benzene ring to a node, called *benzene node*, labeled with a special label ‘b’, and contract a naphthalene ring to two benzene nodes connected by a special bond, called *merge bond*, represented by a double edge (see Fig. 1). Since six carbon atoms contained in a benzene ring are contracted into a benzene node, we need to remember which carbon atom in the benzene ring connects to its adjacent node in a molecular graph. Hence, we add an attribute called *carbon position list* to each benzene node. Figure 1b shows examples of carbon position lists using numbers assigned to carbon atoms in benzene rings in Fig. 1a. We call such a node-labeled rooted ordered tree whose benzene nodes are attributed with carbon position lists *a carbon position-assigned molecular tree*. We enumerate carbon position-assigned molecular trees instead of molecular graphs.

#### Center-rooted and left-heavy

In our previous work, we defined the normal form for molecular trees without any cyclic structures using *center-rooted* and *left-heavy* to avoid its redundant generation. In this work, we also utilize center-rooted and left-heavy for carbon position-assigned molecular trees, of which properties do not depend on carbon position lists.

**Fig. 2:**
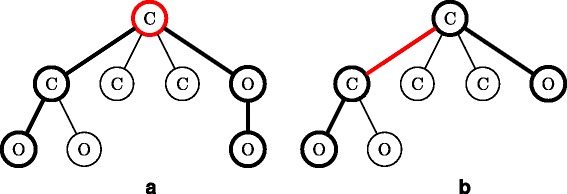
Illustration of center-rooted molecular trees. **a** Center of the longest path is a node. **b** Center of the longest path is an edge. The *thick lines* indicate one of the longest paths and the center node/edge is shown in *red*

A molecular tree *T* is called *center-rooted* if its root is the center node (see Fig. 2a) or one endpoint of the center edge of the longest path in *T* (see Fig. 2b). The center can be either a node or an edge depending on the length of the longest path.

**Fig. 3:**
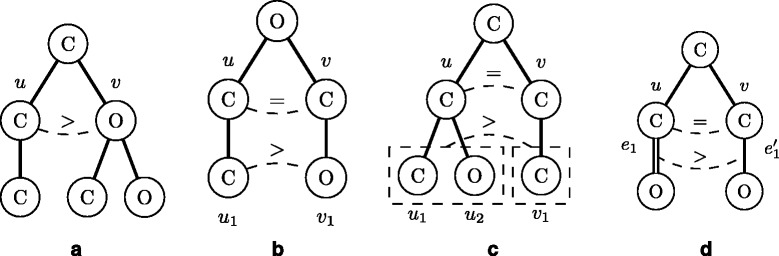
Illustration of three molecular trees such that *T*(*u*)>_*s*_ *T*(*v*) or *T*(*u*)>_*m*_ *T*(*v*). **a** *l*(*u*)>*l*(*v*). **b** *l*(*u*)=*l*(*v*), *T*(*u*_1_)>_*s*_ *T*(*v*_1_). **c** *l*(*u*)=*l*(*v*), *T*(*u*_1_)=_*s*_ *T*(*v*_1_), *h*=2>1=*k*. **d** *T*(*u*)=_*s*_ *T*(*v*), *m* *u* *l*(*e*_1_)>*m* *u* *l*(*e*1′)

In order to define a left-heavy tree, atom-labels must be ordered so that they can be compared with each other, for example, b >*C*>*N*>*O*>H for *Σ*={b,C,N,O,H }, where ‘b’ denotes a special atom representing a benzene ring. Let *T*(*u*) be the ordered subtree rooted at *u* in *T*. Let *u* and *v* be two nodes in a molecular tree *T*, (*u*_1_,*u*_2_,…,*u*_*h*_) and (*v*_1_,*v*_2_,…,*v*_*k*_) be lists of child nodes of *u* and *v*, respectively. It is defined that *T*(*u*)>_*s*_*T*(*v*) if *l*(*u*)>*l*(*v*) (Fig. 3a) or there exists an integer *i* such that *T*(*u*_*j*_)=_*s*_*T*(*v*_*j*_) for all *j*<*i* and (*T*(*u*_*i*_)>_*s*_*T*(*v*_*i*_) (Fig. 3b) or *i*=*k*+1≤*h* (Fig. 3c)). If *T*(*u*)>_*s*_*T*(*v*) or *T*(*v*)>_*s*_*T*(*u*) does not hold, it is said that *T*(*u*)=_*s*_*T*(*v*).

**Fig. 4:**
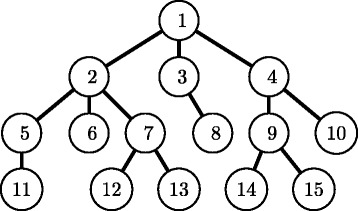
Illustration of breadth-first search (BFS) order. *Numbers* indicate BFS order for this example

Let *mul*(*e*) and *mul*(*u,v*) be the multiplicity of edge *e*=(*u,v*). Let (*e*_1_,*e*_2_,…,*e*_*m*_) and ($e^{\prime }_{1},e'_{2},\ldots,e'_{m}$) be two lists of edges in *T*(*u*) and *T*(*v*) in breadth-first search (BFS) order (see Fig. 4), respectively. *T*(*u*)>_*m*_*T*(*v*) if *T*(*u*)>_*s*_*T*(*v*), or if *T*(*u*)=_*s*_*T*(*v*) and there exists an integer *i* such that *mul*(*e*_*j*_)=*mul*(*ej*′) for all *j*<*i*, and *mul*(*e*_*i*_)>*mul*(*ei*′) (Fig. 3d). If *T*(*u*)>_*m*_*T*(*v*) or *T*(*v*)>_*m*_*T*(*u*) does not hold, it is said that *T*(*u*)=_*m*_*T*(*v*).

Let *child*(*v*)=(*v*_1_,*v*_2_,…) be a list of all child nodes of node *v* in BFS order. It is defined that a molecular tree *T* is *left-heavy* if *T*(*v*_*i*_)≥_*m*_*T*(*v*_*i*+1_) holds for all nodes *v* in *T* and all *i*=1,…,|*child*(*v*)|−1.

It should be noted that center-rooted and left-heavy are different from *centroid-rooted* and *left-heavy* defined by Fujiwara et al. [16], for example, the molecular tree in Fig. 1b is center-rooted and is not centroid-rooted because the number of nodes in the left subtree by removing the root, 4, is more than (total number of nodes −1)/2=(7−1)/2=3. In addition, their left-heavy is defined using depth-first search order, not our breadth-first search order.

#### Carbon position list

**Fig. 5:**
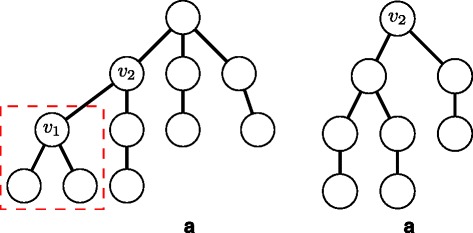
Illustration of subtree *T* ^*sub*^(*v*_1_,*v*_2_). **a** A molecular tree *T* and *T* ^*sub*^(*v*_1_,*v*_2_), which is surrounded by a red rectangle. **b** *T* ^*sub*^(*v*_2_,*v*_1_)

Let *s*=(*v*_1_,*v*_2_,…,*v*_*n*_) be a list of nodes, |*s*| and *s*[ *i*] denote the size and the *i*-th element of *s*, respectively. Let *T*^*sub*^(*v*_1_,*v*_2_) be the left-heavy tree rooted at *v*_1_ that consists of the connected component including *v*_1_ when the edge (*v*_1_,*v*_2_) is deleted from *T* (see Fig. 5). *T*^*sub*^(*v*_1_,*v*_2_)=_*m*_*T*(*v*_1_) if *v*_1_ is a child of *v*_2_ in *T*. Let *index*(*v,T*) be the order of *v*∈*V*(*T*) by traversing a center-rooted left-heavy molecular tree *T* with BFS order, which is also denoted by *index*(*v*) if *T* is clear.

##### **Proposition1**.

For a node *v* that has the parent node *v*_*p*_ and a child node *v*_*c*_ in a center-rooted molecular tree *T*, *T*^*sub*^(*v*_*p*_,*v*)≠_*m*_*T*^*sub*^(*v*_*c*_,*v*).

##### *Proof*.

The height of *T*^*sub*^(*v*_*p*_,*v*) is larger than that of *T*^*sub*^(*v*_*c*_,*v*) because *T* is center-rooted. Hence, *T*^*sub*^(*v*_*p*_,*v*) is always different from *T*^*sub*^(*v*_*c*_,*v*).

We define an equality *T*_1_=_*C*_*T*_2_ for two rooted carbon-position assigned trees *T*_1_ and *T*_2_ if *T*_1_=_*m*_*T*_2_, and *Cv*_1_*T*_1_=*Cv*_2_*T*_2_ for all benzene nodes *v*_1_∈*V*(*T*_1_), where *v*_2_∈*V*(*T*_2_) satisfies *index*(*v*_1_,*T*_1_)=*index*(*v*_2_,*T*_2_), and ${C^{T}_{v}}$ is a list of lists, called a carbon position list explained later, for a benzene node *v* in *T*. For convenience, we define another equality $T_{1}=_{\underline {C}}T_{2}$ by removing the condition that *Cr*_1_*T*_1_=*Cr*_2_*T*_2_ for the roots *r*_1_ and *r*_2_ of *T*_1_ and *T*_2_, respectively, from the conditions of *T*_1_=_*C*_*T*_2_, if *r*_1_ and *r*_2_ are benzene nodes.

For a node *v* having the parent *v*_*p*_ and a child *v*_*c*_, *T*^*sub*^(*v*_*p*_,*v*)≠_*C*_*T*^*sub*^(*v*_*c*_,*v*) if *T*^*sub*^(*v*_*p*_,*v*)≠_*m*_*T*^*sub*^(*v*_*c*_,*v*). Hence, only carbon position lists of descendent benzene nodes are needed to determine whether or not $T^{sub}(v_{c_{1}},v)=_{C} T^{sub}(v_{c_{2}},v)$ for child nodes $v_{c_{1}}$ and $v_{c_{2}}$ of *v*.

##### **Definition1**.

An *adjacent node list*${A^{T}_{v}}$ of a benzene node *v* in a carbon position-assigned molecular tree *T* is defined as a list of lists of nodes adjacent to *v* using carbon position lists of descendent benzene nodes such that

- $|{A^{T}_{v}}[\!i]|\leq |{A^{T}_{v}}[\!i+1]|$ for all *i*,
- $index \left ({A^{T}_{v}}[\!i][\!1]\right) < index \left ({A^{T}_{v}}[\!i+1][\!1]\right)$ if $|{A^{T}_{v}}[\!i]|=|{A^{T}_{v}}[\!i+1]|$,
- $index \left ({A^{T}_{v}}[\!i][\!j]\right) < index \left ({A^{T}_{v}}[\!i][j+1]\right)$ for all *i*,*j*,
- ${A^{T}_{v}}[\!i]=(v')$ if (*v, v*^′^) is a merge bond for some *i*,
- $v'\in {A_{v}^{T}}[\!i]$ if (*v, v*^′^) is not a merge bond, and $T^{sub}(v',v)=_{C}T^{sub} \left ({A^{T}_{v}}[\!i][\!1],v\right)$.

**Fig. 6:**
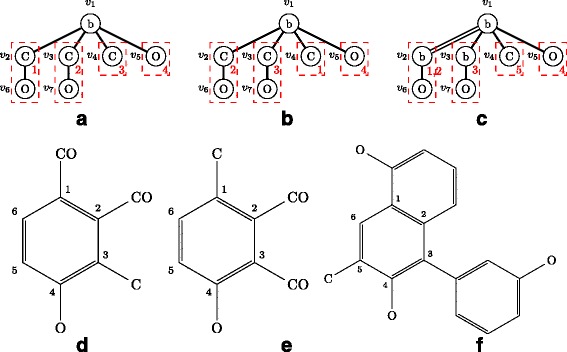
Examples of adjacent node lists and carbon position lists. **a** *T*_1_. **b** *T*_2_. **c** *T*_3_. **d** Molecular graph of *T*_1_. **e** Molecular graph of *T*_2_. **f** Molecular graph of *T*_3_. Red numbers represent carbon positions of node *v*_1_

Figure 6 shows examples of carbon position-assigned molecular trees, where benzene node *v*_1_ in each tree has adjacent nodes *v*_2_,*v*_3_,*v*_4_,*v*_5_. Then, $T_{1}^{sub}(v_{2},v_{1}) =_{C} T_{1}^{sub}(v_{3},v_{1}) \neq _{C} T_{1}^{sub}(v_{4},v_{1}) \neq _{C} T_{1}^{sub}(v_{5},v_{1})$ and *index*(*v*_4_)<*index*(*v*_5_), so we have $A^{T_{1}}_{v_{1}}=((v_{4}),(v_{5}),(v_{2},v_{3}))$. Also for *T*_2_, $A^{T_{2}}_{v_{1}}=((v_{4}),(v_{5}),(v_{2},v_{3}))$. For *T*_3_, $A^{T_{3}}_{v_{1}}=((v_{2}),(v_{3}),(v_{4}),(v_{5}))$ because (*v*_2_,*v*_1_) is a merge bond. If (*v*_2_,*v*_1_) is not a merge bond and *Cv*_2_*T*_3_=*Cv*_3_*T*_3_, then $A^{T_{3}}_{v_{1}}=((v_{4}),(v_{5}),(v_{2},v_{3}))$.

##### **Proposition2**.

For a benzene node *v* that has the parent node *v*_*p*_ in a center-rooted molecular tree *T*, ${A^{T}_{v}}[\!1]=(v_{p})$.

##### *Proof*.

If *v* has no child, it is clear because the adjacent node of *v* is only *v*_*p*_. We assume that *v* has a child *v*_*c*_. From Proposition 1 and *index*(*v*_*p*_)<*index*(*v*_*c*_), ${A^{T}_{v}}[\!1]=(v_{p})$ always holds.

A *carbon position list*${C^{T}_{v}}$ of a benzene node *v* in *T* is a list of lists, where ${C^{T}_{v}}[\!i]$ is a list of carbon positions of the nodes in ${A^{T}_{v}}[\!i]$. It is sufficient to enumerate ${C^{T}_{v}}[\!i]$ in ascending order because each node in ${A^{T}_{v}}[\!i]$ has the same subtree. If $\left ({A^{T}_{v}}[\!i][\!1],v\right)$ is a merge bond, ${C^{T}_{v}}[\!i]$ has two carbon positions instead of one as usual. It should be noted that ${C^{T}_{v}}[\!i] \subseteq \{1,\ldots,6\}$ and two carbon positions are assigned for a merge bond because a naphthalene ring shares two carbon atoms between two benzene rings. In the examples of Fig. 6, $C^{T_{1}}_{v_{1}}=((3),(4),(1,2))$ for $A^{T_{1}}_{v_{1}}=((v_{4}),(v_{5}),(v_{2},v_{3}))$, $C^{T_{2}}_{v_{1}}=((1),(4),(2,3))$ for $A^{T_{2}}_{v_{1}}=((v_{4}),(v_{5}),(v_{2},v_{3}))$, $C^{T_{3}}_{v_{1}}=((1,2),(3),(5),(4))$ for $A^{T_{3}}_{v_{1}}=((v_{2}),(v_{3}),(v_{4}),(v_{5}))$.

##### **Definition2**.

An *adjacent node list*$A^{T}_{(v_{1},v_{2})}$ for a naphthalene ring with two benzene nodes *v*_1_, *v*_2_, where (*v*_1_,*v*_2_) is a merge bond, is defined as a list of lists of nodes adjacent to *v*_1_ or *v*_2_ except *v*_1_ and *v*_2_ such that

- $|A^{T}_{(v_{1},v_{2})}[\!i]|\leq |A^{T}_{(v_{1},v_{2})}[\!i+1]|$ for all *i*,
- $index \left (A^{T}_{(v_{1},v_{2})}[\!i][\!1]\right) < index \left (A^{T}_{(v_{1},v_{2})}[\!i+1][\!1]\right)$ if $|A^{T}_{(v_{1},v_{2})}[\!i]|=|A^{T}_{(v_{1},v_{2})}[\!i+1]|$,
- $index \left (A^{T}_{(v_{1},v_{2})}[\!i][\!j]\right) < index \left (A^{T}_{(v_{1},v_{2})}[\!i][j+1]\right)$ for all *i*,*j*,
- $v'\in A^{T}_{(v_{1},v_{2})}[\!i]$ if $T^{sub}(v',bn(v'))=_{C}T^{sub} \left (A^{T}_{(v_{1},v_{2})}[\!i][\!1],bn(A^{T}_{(v_{1},v_{2})}[\!i][\!1])\right)$, where *bn*(*v*) is *v*_1_ or *v*_2_ that is adjacent to *v*.

**Fig. 7:**
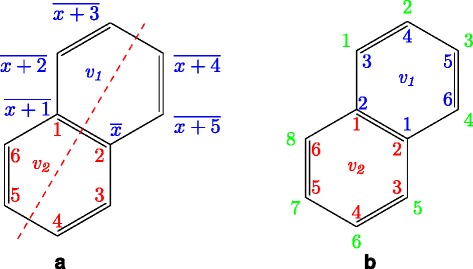
Correspondence between carbon positions in a naphthalene ring. **a** Correspondence between carbon positions involved with a merge bond in two benzene rings. **b** Correspondence between carbon positions of a naphthalene ring and two benzene rings in the case of $T^{sub}(v_{1},v_{2})=_{\underline {C}}T^{sub} (v_{2},v_{1})$. The upper benzene ring *v*_1_ is the parent of the lower benzene ring *v*_2_. $\overline {x}$ denotes (*x* mod 6)+1. Blue, red, and green numbers are positions of $C^{T}_{v_{1}}$, $C^{T}_{v_{2}}$, and $C^{T}_{(v_{1},v_{2})}$, respectively. The dashed red line denotes the symmetric axis of *ϕ*_*ref*_

For a benzene node *v*_2_ that is connected by a merge bond with the parent node *v*_1_, we suppose that the carbon atoms having positions 1,2 in *v*_2_ are connected with the carbon atoms having positions $\overline {x+1}, \overline {x}$ in *v*_1_, respectively, where *x* takes an integer between 1 and 6, and $\overline {x}=(x \mod 6)+1$ (see Fig. 7a). Here, consider the case that *v*_1_ has the parent node *v*_*p*_. If *T* is in normal form (Definition 6), position 1 is assigned to the carbon atom connected with *v*_*p*_ (Proposition 5). Then, from Proposition 1, *T*^*sub*^(*v*_*p*_,*v*_1_)≠_*C*_*T*^*sub*^(*v*_*c*_,*v*_2_) for any child node *v*_*c*_ of *v*_2_, *T*^*sub*^(*v*_*p*_,*v*_1_)≠_*C*_*T*^*sub*^(*v*_*c*_,*v*_1_) for any child node *v*_*c*_ of *v*_1_ except *v*_2_, and the naphthalene ring is not symmetric. Consider the case that *v*_1_ does not have a parent node, that is, *v*_1_ is the root. If $T^{sub}(v_{1},v_{2})\neq _{\underline {C}}T^{sub}(v_{2},v_{1})$, the naphthalene ring can be symmetric only with respect to the axis denoted by the dashed red line in Fig. 7a. Then, it is not needed to consider the other symmetry for the naphthalene ring.

Consider the case that $T^{sub}(v_{1},v_{2})=_{\underline {C}}T^{sub}(v_{2},v_{1})$. We can prove that *x*=1 if *T* is in normal form (see Proposition 4). Then, a carbon position list $C^{T}_{(v_{1}, v_{2})}$ of a naphthalene ring consisting of two benzene nodes *v*_1_, *v*_2_ is a list of lists determined from $C^{T}_{v_{1}}$ and $C^{T}_{v_{2}}$ according to the following rule, where $C^{T}_{(v_{1}, v_{2})}[\!i]$ is a list of carbon positions of nodes in $A^{T}_{(v_{1}, v_{2})}[\!i]$ in ascending order.

##### **Definition3**.

Carbon positions in a naphthalene ring correspond to carbon positions in two benzene nodes *v*_1_,*v*_2_, where *v*_1_ is the parent node of *v*_2_, if $T^{sub}(v_{1},v_{2})=_{\underline {C}}T^{sub}(v_{2},v_{1})$, as follows (see Fig. 7b).

- For the benzene ring of *v*_1_, positions 1,2 are assigned to carbons of the merge bond in $C^{T}_{v_{1}}$. Position *i* (*i*=3,…,6) in $C^{T}_{v_{1}}$ corresponds to *i*−2 in $C^{T}_{(v_{1}, v_{2})}$.
- For the benzene ring of *v*_2_, positions 1,2 are assigned to carbons of the merge bond in $C^{T}_{v_{2}}$. Position *i* (*i*=3,…,6) in $C^{T}_{v_{2}}$ corresponds to *i*+2 in $C^{T}_{(v_{1}, v_{2})}$.

**Fig. 8:**
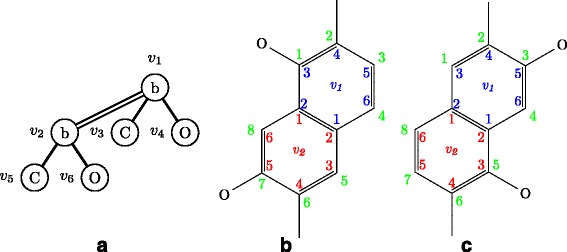
Example of carbon position lists for a naphthalene ring. **a** *T*_4_. **b** Molecular graph of $T^{\prime }_{4}$, which is *T*_4_ with $C^{T'_{4}}_{v_{1}}=((1,2),(4),(3))$, $C^{T'_{4}}_{v_{2}}=((1,2), (4),(5))$. **c** Molecular graph of $T^{\prime \prime }_{4}$, which is *T*_4_ with $C^{T^{\prime \prime }_{4}}_{v_{1}}=((1,2),(4),(5))$, and $C^{T^{\prime \prime }_{4}}_{v_{2}}=((1,2),(4),(3))$

Figure 8 shows examples of carbon position lists for a naphthalene ring, where $T^{\prime }_{4}$ is *T*_4_ with $C^{T'_{4}}_{v_{1}}=((1,2),(4),(3))$ and $C^{T'_{4}}_{v_{2}}=((1,2),(4),(5))$, $T^{\prime \prime }_{4}$ is *T*_4_ with $C^{T^{\prime \prime }_{4}}_{v_{1}}=((1,2),(4),(5))$ and $C^{T^{\prime \prime }_{4}}_{v_{2}}=((1,2),(4),(3))$. Then, $A^{T'_{4}}_{(v_{1},v_{2})}=A^{T^{\prime \prime }_{4}}_{(v_{1},v_{2})}=((v_{3},v_{5}),(v_{4},v_{6}))$, $C^{T'_{4}}_{(v_{1},v_{2})}=((2,6),(1,7))$, and $C^{T^{\prime \prime }_{4}}_{(v_{1},v_{2})}=((2,6),(3,5))$.

##### **Definition4**.

For carbon position lists $C^{T_{1}}_{v}$, $C^{T_{2}}_{v}$, where *AvT*_1_=*AvT*_2_, it is defined that *CvT*_1_<*CvT*_2_ if there exist two integers *i* and *j* such that

- *CvT*_1_[ *i*^′^][*j*^′^]=*CvT*_2_[ *i*^′^][*j*^′^] for all *i*^′^<*i* and all $j' = 1,\ldots,|C^{T_{1}}_{v}[\!i']|$,
- *CvT*_1_[ *i*][*j*^′^]=*CvT*_2_[ *i*][*j*^′^] for all *j*^′^<*j*,
- *CvT*_1_[ *i*][ *j*]<*CvT*_2_[ *i*][ *j*].

This definition is applied to comparison of $C^{T_{1}}_{(v_{1},v_{2})}$ and $C^{T_{2}}_{(v_{1},v_{2})}$ for a naphthalene ring with *v*_1_ and *v*_2_ in the same way.

In the example of Fig. 6, *T*_1_ and *T*_2_ have the same tree structure, and *Cv*_1_*T*_2_=((1),(4),(2,3))<((3),(4),(1,2))=*Cv*_1_*T*_1_ because *Cv*_1_*T*_2_[ 1][ 1]=1<3=*Cv*_1_*T*_1_[ 1][ 1].

**Fig. 9:**
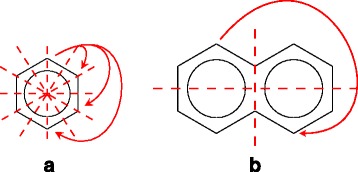
Illustration of automorphism of a benzene ring and a naphthalene ring. **a** A benzene ring. **b** A naphthalene ring. *Dashed lines* indicate reflections, curves indicate rotations, where all automorphisms are not shown

Let *Aut*_*b*_ and *Aut*_*n*_ be the automorphism groups of a benzene ring and a naphthalene ring, respectively (see Fig. 9). *Aut*_*b*_ is generated from rotation of *π*/3 radians and reflection. For *ϕ*_*b*_∈*Aut*_*b*_, *v*_1_ is adjacent to *v*_2_ in a benzene ring if and only if *ϕ*_*b*_(*v*_1_) is adjacent to *ϕ*_*b*_(*v*_2_) in a benzene ring. *Aut*_*n*_ is generated from rotation of *π* radians and reflection. We suppose that a list $\phi ({C^{T}_{v}}[\!i])$ of carbon positions for a map *ϕ* and $i=1,\dots,|{C^{T}_{v}}|$ is in ascending order by sorting elements of the list because all nodes in ${A^{T}_{v}}[\!i]$ have the same subtree. For example, *ϕ*_*b*_(*Cv*_1_*T*_1_)=((6),(5),(1,2)) for $C^{T_{1}}_{v_{1}}=((3),(4),(1,2))$ and the reflection map *ϕ*_*b*_ by the perpendicular bisector between carbon atoms of 1 and 2.

### Normal form of a carbon position-assigned molecular tree

In order to prevent generating redundant molecular trees in enumeration, we define a normal form of a carbon position-assigned molecular tree.

#### **Definition5**.

Let *P* be a path in *T* consisting of *n* nodes (*v*_1_,*v*_2_,…,*v*_*n*_)(*n*≥2). *P* is called a *symmetric path* if the following conditions are satisfied.

- $T^{sub} \left (v_{\lfloor \frac {n}2\rfloor },v_{\lfloor \frac {n}2\rfloor +1}\right) {=\;}_{m}T^{sub} \left (v_{n-\lfloor \frac {n}2\rfloor +1},v_{n-\lfloor \frac {n}2\rfloor }\right)$,
- $index \left (v_{i},T^{sub}\left (v_{\lfloor \frac {n}2\rfloor },v_{\lfloor \frac {n}2\rfloor +1}\right)\right) = index \left (v_{n-i+1},T^{sub}\left (v_{n-\lfloor \frac {n}2\rfloor +1},v_{n-\lfloor \frac {n}2\rfloor }\right)\right)$ for all $i=1,\cdots,\lfloor \frac {n}2\rfloor $, where ⌊*x*⌋ is the largest integer less than or equal to *x*,
- ${C^{T}_{v}} = C^{T}_{v'}$ for all benzene nodes $v\in V\left (T^{sub}\left (v_{\lfloor \frac {n}2\rfloor },v_{\lfloor \frac {n}2\rfloor +1}\right)\right) \backslash V \left (T^{sub}(v_{1},v_{2})\right)$, where $v'\in V \left (T^{sub}\left (v_{n-\lfloor \frac {n}2\rfloor +1},v_{n-\lfloor \frac {n}2\rfloor }\right)\right)$ satisfies $index \left (v', T^{sub}\left (v_{n-\lfloor \frac {n}2\rfloor +1},v_{n-\lfloor \frac {n}2\rfloor }\right)\right) = index \left (v, T^{sub}\left (v_{\lfloor \frac {n}2\rfloor },v_{\lfloor \frac {n}2\rfloor +1}\right)\right)$, and *v*∈*V*_1_∖*V*_2_ means that *v*∈*V*_1_ and *v*∉*V*_2_.

#### **Proposition3**.

For a center-rooted molecular tree, either of $v_{\frac {n}2}$ and $v_{\frac {n}2+1}$ is the root if the length of a symmetric path (*v*_1_,⋯,*v*_*n*_) is even. Otherwise, the depth of $v_{\frac {n+1}2}$ is less than that of any node in the path.

#### *Proof*.

For a path (*v*_1_,⋯,*v*_*n*_), *v*_*i*+1_ and *v*_*n*−*i*_ must be the parent nodes of *v*_*i*_ and *v*_*n*−*i*+1_, respectively, for $i=1,\cdots,\frac {n-1}{2}$ if *n* is odd and for $i=1,\cdots,\frac {n}{2}-1$ if *n* is even due to the center rooted property. Therefore, if the length of path is odd, $v_{\frac {n+1}{2}}$ is the parent node of both $v_{\frac {n+1}{2}-1}$ and $v_{\frac {n+1}{2}+1}$, which means that the depth of $v_{\frac {n+1}2}$ is less than that of any node in the path.

In the case that *n* is even, either $v_{\frac {n}{2}}$ or $v_{\frac {n}{2}+1}$ has the least depth among all nodes in the path and another node is the child node of that node. Assume that between these two nodes the parent node is *v*_*a*_ and the child node is *v*_*b*_. *v*_*a*_ cannot have a parent node because the height of *T*^*sub*^(*v*_*p*_,*v*_*a*_), where *v*_*p*_ is the parent node of *v*_*a*_, cannot be equal to the height of *T*^*sub*^(*v*_*c*_,*v*_*b*_) for any nodes *v*_*c*_ that are adjacent to *v*_*b*_ due the center-rooted condition, which means that *T*^*sub*^(*v*_*a*_,*v*_*b*_)=_*m*_*T*^*sub*^(*v*_*b*_,*v*_*a*_) cannot be hold and the first condition of symmetric path is violated. In other words, *v*_*a*_, which is either $v_{\frac {n}{2}}$ or $v_{\frac {n}{2}+1}$, is the root node of the tree if *n* is even.

We say that *v*_1_ is *left* of *v*_*n*_ for a symmetric path (*v*_1_,…,*v*_*n*_) when $v_{n-\lfloor \frac {n}2\rfloor +1}$ is the root, or *index*(*v*_1_)<*index*(*v*_*n*_).

**Fig. 10:**
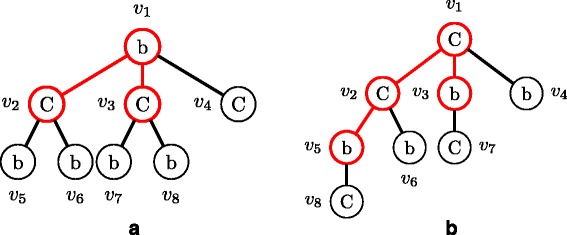
Examples of symmetric paths. The *red lines* denote symmetric paths. **a** *T*_5_, where (*v*_2_,*v*_1_,*v*_3_) is a symmetric path, and $T_{5}^{sub}(v_{2},v_{1})=_{m}T_{5}^{sub} (v_{3},v_{1})$. **b** *T*_6_, where (*v*_5_,*v*_2_,*v*_1_,*v*_3_) is a symmetric path, $T_{6}^{sub}(v_{2},v_{1})=_{m}T_{6}^{sub}(v_{1},v_{2})$ and *C* *v*_4_ *T*_6_=*C* *v*_6_ *T*_6_

Figure 10 shows examples of symmetric paths, (*v*_2_,*v*_1_,*v*_3_) in *T*_5_ and (*v*_5_,*v*_2_,*v*_1_,*v*_3_) in *T*_6_, where $T_{5}^{sub}(v_{2},v_{1})=_{m}T_{5}^{sub}(v_{3},v_{1})$, $T_{6}^{sub}(v_{2},v_{1})=_{m}T_{6}^{sub}(v_{1},v_{2})$, and *Cv*_4_*T*_6_=*Cv*_6_*T*_6_.

We define an inequality *T*_1_>_*C*_*T*_2_ for carbon position-assigned molecular trees *T*_1_ and *T*_2_ if *T*_1_>_*m*_*T*_2_, or *T*_1_=_*m*_*T*_2_, and there exists an integer *i* such that *v*_*i*_ is a benzene node, *Cv*_*i*_*T*_1_>*Cvi*′*T*_2_, and *Cv*_*j*_*T*_1_=*Cvj*′*T*_2_ for all benzene nodes *v*_*j*_ with *j*>*i*, where *index*(*v*_*k*_,*T*_1_)=*index*(*vk*′,*T*_2_) for all *k*=1,…,|*V*(*T*_1_)|.

#### **Definition6**.

Let *ϕ*_*ref*_ be the reflection map with the symmetric axis shown in Fig. 7a. A carbon position-assigned molecular tree *T* that contains a carbon position list ${C^{T}_{v}}$ for each benzene node *v* is in *normal form* if the following conditions are satisfied.

1. *T* is center-rooted and left-heavy.
2. *T*(*v*)≥_*m*_*T*^*sub*^(*r*,*v*) if the center of the longest path in *T* with the root *r* is the edge (*r*,*v*).
3. Positions in each sublist of ${C^{T}_{v}}$ for each benzene node *v* are in ascending order.
4. ${C^{T}_{v}}\leq \phi _{b}\left ({C^{T}_{v}}\right)$ for all benzene nodes *v* that is not connected by a merge bond with the parent node and all *ϕ*_*b*_∈*Aut*_*b*_.
5. For benzene nodes *v*_1_,*v*_2_ connected by a merge bond such that *v*_1_ is the root of *T*,
  - $C^{T}_{(v_{1},v_{2})}\leq \phi _{n} \left (C^{T}_{(v_{1},v_{2})}\right)$ for all *ϕ*_*n*_∈*Aut*_*n*_ if $T^{sub}(v_{1},v_{2})=_{\underline {C}}T^{sub}(v_{2},v_{1})$, where $C^{T}_{(v_{1},v_{2})}$ is related with $C^{T}_{v_{1}}$ and $C^{T}_{v_{2}}$ by Definition 3.
  - $C^{T}_{v_{2}}\leq \phi_{ref} \left (C^{T}_{v_{2}}\right)$ if $T^{sub}(v_{1},v_{2})\neq _{\underline {C}} T^{sub}(v_{2},v_{1})$ and $C^{T}_{v_{1}}=\phi_{ref} \left (C^{T}_{v_{1}}\right)$.
6. *T*^*sub*^(*v*_1_,*v*_2_)≥_*C*_*T*^*sub*^(*v*_*n*_,*v*_*n*−1_) for all pairs *v*_1_,*v*_*n*_ of nodes such that the path (*v*_1_,…,*v*_*n*_) is a symmetric path, *v*_1_ and *v*_*n*_(=*v*_2_) are not connected by a merge bond, and *v*_1_ is left of *v*_*n*_.

We call a tree in normal form a *normal tree*.

Figure 8 also shows molecular trees in normal form and not in normal form. For condition 4 of the definition, $C^{T'_{4}}_{v_{1}}=((1,2),(4),(3))\leq \phi _{b} \left (C^{T'_{4}}_{v_{1}}\right)$, $C^{T^{\prime \prime }_{4}}_{v_{1}} = ((1,2),(4),(5))\leq \phi _{b}\left (C^{T^{\prime \prime }_{4}}_{v_{1}}\right)$. $T^{\prime }_{4}$ and $T^{\prime \prime }_{4}$ satisfy conditions 1, 2, 3, and 4. For condition 5, $C^{T'_{4}}_{(v_{1},v_{2})}=((2,6),(1,7))\leq \phi _{n}\left (C^{T'_{4}}_{(v_{1},v_{2})}\right)$, whereas $C^{T^{\prime \prime }_{4}}_{(v_{1},v_{2})}=((2,6),(3,5))>((2,6),(1,7))=\phi_{rot}\left (C^{T^{\prime \prime }_{4}}_{(v_{1},v_{2})}\right)$ for rotation *ϕ*_*rot*_ of *π* radians, and $T^{\prime \prime }_{4}$ violates the condition. It is noted that $T^{\prime \prime }_{4}$ is rotated by *π* radians from $T^{\prime }_{4}$. For condition 6, *v*_1_ and *v*_2_ are connected by a merge bond. Thus, $T^{\prime }_{4}$ is a normal tree, and $T^{\prime \prime }_{4}$ is not a normal tree.

#### **Proposition4**.

For a normal tree *T* with a benzene node *v*_1_ that is connected by a merge bond with its child node *v*_2_ and satisfies $T^{sub}(v_{1},v_{2})=_{\underline {C}}T^{sub}(v_{2},v_{1})$, positions 1,2 are assigned to the merge bond in the benzene ring of *v*_1_. Furthermore, if $C^{T}_{(v_{1},v_{2})}\leq \phi _{n}\left (C^{T}_{(v_{1},v_{2})}\right)$ for all *ϕ*_*n*_∈*Aut*_*n*_, then $C^{T}_{v_{1}}\leq \phi _{b}\left (C^{T}_{v_{1}}\right)$ for all *ϕ*_*b*_∈*Aut*_*b*_.

#### *Proof*.

We assume that there exists a node *v*_*l*_ as a left sibling of *v*_2_, and *v*_*l*_ is the leftmost child of *v*_1_. Since *T* is left-heavy, *T*(*v*_*l*_)≥_*m*_*T*(*v*_2_), and *l*(*v*_*l*_)=*l*(*v*_2_)=‘b’ is needed. However, *T*(*v*_*l*_)=_*C*_*T*(*v*_*c*_), where *v*_*c*_ is the leftmost child of *v*_2_, because $T^{sub}(v_{1},v_{2})=_{\underline {C}}T^{sub}(v_{2},v_{1})=_{C}T(v_{2})$. Hence, *T*(*v*_*l*_)<_*m*_*T*(*v*_2_). It contradicts the assumption, and *v*_2_ is the leftmost child of *v*_1_. Therefore, $A^{T}_{v_{1}}[\!1]=(v_{2})$. From condition 4 of Definition 6, $C^{T}_{v_{1}}[\!1]=(1,2)$, and positions 1,2 are assigned to the merge bond, that is *x*=1 in Fig. 7a.

For a map *ϕ*_*b*_∈*Aut*_*b*_ other than the identity and reflection map *ϕ*_*ref*_ for a benzene ring, $C^{T}_{v_{1}}<\phi _{b}\left (C^{T}_{v_{1}}\right)$ because each of *ϕ*_*b*_(1) and *ϕ*_*b*_(2) is at least 2. From $C^{T}_{(v_{1},v_{2})}\leq \phi_{ref}\left (C^{T}_{(v_{1},v_{2})}\right)$ and the correspondence between $C^{T}_{v_{1}}$ and $C^{T}_{(v_{1},v_{2})}$, $C^{T}_{v_{1}}\leq \phi_{ref}\left (C^{T}_{v_{1}}\right)$. Therefore, $C^{T}_{v_{1}}\leq \phi _{b}\left (C^{T}_{v_{1}}\right)$ for all *ϕ*_*b*_∈*Aut*_*b*_.

#### **Proposition5**.

For a benzene node *v* of a normal tree *T*, ${C^{T}_{v}}[\!1][\!1]$ is always equal to 1.

#### *Proof*.

If *v* is not connected by a merge bond with the parent node, from condition 4, ${C^{T}_{v}}$ must be the least possible carbon position list. Hence, ${C^{T}_{v}}[\!1][\!1]=1$. Otherwise, from Definition 3, ${C^{T}_{v}}[\!1][\!1]=1$.

#### **Lemma1**.

Given a molecular graph *G* without cyclic structures except benzene rings and naphthalene rings, *G* can be represented by a normal tree.

#### *Proof*.

We can assign numbers to carbons in benzene rings and naphthalene rings of *G* such that the conditions of Definition 6 are satisfied.

#### **Lemma2**.

Given two different molecular graphs *G*_1_ and *G*_2_, they cannot be represented by the same normal tree.

#### *Proof*.

We can unambiguously obtain a molecular graph from a normal tree by replacing all benzene nodes with benzene rings according to its carbon position lists.

#### **Proposition6**.

For a normal tree *T* with a path (*v*_1_,…,*v*_*n*_), *G*^′^ is the molecular graph obtained from the tree *T*^′^ by removing *T*^*sub*^(*v*_1_,*v*_2_) and *T*^*sub*^(*v*_*n*_,*v*_*n*−1_) except *v*_1_ and *v*_*n*_ from *T*, where *v*_1_ is left of *v*_*n*_. If there is a non-identity map *ϕ* of the automorphism group of *G*^′^ satisfying *ϕ*(*v*_*i*_)=*v*_*n*−*i*+1_ for all *i*=1,…,*n*, then *T*^*sub*^(*v*_1_,*v*_2_)≥_*C*_*T*^*sub*^(*v*_*n*_,*v*_*n*−1_), where *ϕ* in *G*^′^ is naturally extended to *T*.

#### *Proof*.

**Fig. 11:**
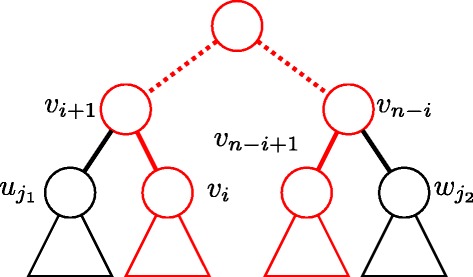
Illustration of an automorphism *ϕ* in the proof. The *red path* indicates (*v*_1_,…,*v*_*n*_), where *ϕ*(*v*_*i*_)=*v*_*n*−*i*+1_ for all *i*=1,…,*n*

If $T^{sub}\left (\!v_{\lfloor \frac {n}2\rfloor },v_{\lfloor \frac {n}2\rfloor +1}\right)\!\!>_{m} \!T^{sub}\left (v_{n-\lfloor \frac {n}2\rfloor +1},v_{n-\lfloor \frac {n}2\rfloor }\right)$, then *T*^*sub*^(*v*_1_,*v*_2_)>_*m*_*T*^*sub*^(*v*_*n*_,*v*_*n*−1_), and *T*^*sub*^(*v*_1_,*v*_2_)>_*C*_*T*^*sub*^(*v*_*n*_,*v*_*n*−1_). We assume $T^{sub}\left (v_{\lfloor \frac {n}2\rfloor },v_{\lfloor \frac {n}2\rfloor +1}\right)=_{m} T^{sub}\left (v_{n-\lfloor \frac {n}2\rfloor +1},v_{n-\lfloor \frac {n}2\rfloor }\right)$. If the path (*v*_1_,…,*v*_*n*_) is a symmetric path, *T*^*sub*^(*v*_1_,*v*_2_)≥_*C*_*T*^*sub*^(*v*_*n*_,*v*_*n*−1_) from condition 6. We assume that (*v*_*i*+1_,…,*v*_*n*−*i*_) is a symmetric path for some *i*, and *index*(*v*_*i*_,*T*^*sub*^(*v*_*i*+1_,*v*_*i*+2_))>*index*(*v*_*n*−*i*+1_,*T*^*sub*^(*v*_*n*−*i*_,*v*_*n*−*i*−1_)) (see Fig. 11). Then,

(1) $$ \begin{aligned} T^{sub}(v_{i+1},v_{i+2})&=_{m}T^{sub}(v_{n-i},v_{n-i-1}),\\ T^{sub}(v_{i+1},v_{i+2})&\geq_{C}T^{sub}(v_{n-i},v_{n-i-1}).  \end{aligned}  $$

Let *u*_*j*_ and *w*_*j*_ be child nodes of *v*_*i*+1_ and *v*_*n*−*i*_, respectively. Then, $v_{i}=u_{j_{2}\phantom {\dot {i}\!}}$ and $v_{n-i+1}=w_{j_{1}\phantom {\dot {i}\!}}$, where *j*_1_=*index*(*v*_*n*−*i*+1_,*T*^*sub*^(*v*_*n*−*i*_,*v*_*n*−*i*−1_)) and *j*_2_=*index*(*v*_*i*_,*T*^*sub*^(*v*_*i*+1_,*v*_*i*+2_)). If *v*_*i*+1_ and *v*_*n*−*i*_ are benzene nodes, $T(u_{j_{1}})=_{C}T(v_{i})$, $T(v_{n-i+1})=_{C}T\left (w_{j_{2}}\right)$, and *T*(*v*_*i*_)=_*C*_*T*(*v*_*n*−*i*+1_) because $C^{T}_{v_{i+1}}=C^{T}_{v_{n-i}}$ and *ϕ*(*v*_*i*_)=*v*_*n*−*i*+1_.

We assume that *v*_*i*+1_ and *v*_*n*−*i*_ are not benzene nodes. For child nodes *u*_*j*_ of *v*_*i*+1_, *T*(*u*_*j*_)≥_*C*_*T*(*u*_*j*+1_) because (*u*_*j*_,*v*_*i*+1_,*u*_*j*+1_) is a symmetric path. Also for child nodes *w*_*j*_ of *v*_*n*−*i*_, *T*(*w*_*j*_)≥_*C*_*T*(*w*_*j*+1_). From the definition of *ϕ*, *T*(*u*_*j*_)=_*C*_*T*(*ϕ*(*u*_*j*_)) for all *u*_*j*_≠*v*_*i*_. If *index*(*ϕ*(*u*_*j*+*l*_))<*index*(*ϕ*(*u*_*j*_)) for *u*_*j*_,*u*_*j*+*l*_≠*v*_*i*_ and *l*>0, *T*(*u*_*j*_)≥_*C*_*T*(*u*_*j*+*l*_)=_*C*_*T*(*ϕ*(*u*_*j*+*l*_))≥_*C*_*T*(*ϕ*(*u*_*j*_))=_*C*_*T*(*u*_*j*_). It means *T*(*u*_*j*_)=_*C*_*T*(*u*_*j*+*l*_). We assume that *index*(*ϕ*(*u*_*j*_))<*index*(*ϕ*(*u*_*j*+*l*_)) for all *u*_*j*_≠*v*_*i*_, that is, *ϕ*(*u*_*j*_)=*w*_*j*+1_ for all *j*=*j*_1_,…,*j*_2_−1. Then,

(2) $$ \begin{aligned} T\left(u_{j}\right)\,&{=}_{C}T\left(w_{j+1}\right)\leq_{C} T\left(w_{j}\right),\\ \text{and}\ \ T(v_{i})\,&{\leq}_{C} T\left(u_{j_{2}-1}\right){=}_{C} T\left(w_{j_{2}}\right). \end{aligned}  $$

If *T*^*sub*^(*v*_*i*+1_,*v*_*i*+2_)>_*C*_*T*^*sub*^(*v*_*n*−*i*_,*v*_*n*−*i*−1_), then there is an integer *j* (*j*_1_≤*j*≤*j*_2_) such that *T*(*u*_*j*_)>_*C*_*T*(*w*_*j*_), and it contradicts Eq. (2). Therefore, *T*^*sub*^(*v*_*i*+1_,*v*_*i*+2_)=_*C*_*T*^*sub*^(*v*_*n*−*i*_,*v*_*n*−*i*−1_), and *T*(*v*_*i*_)=_*C*_*T*(*v*_*n*−*i*+1_). Also for the case that (*v*_*i*+1_,…,*v*_*n*−*i*_) is a symmetric path for some *i* and *index*(*v*_*i*_,*T*^*sub*^(*v*_*i*+1_,*v*_*i*+2_))<*index*(*v*_*n*−*i*+1_,*T*^*sub*^(*v*_*n*−*i*_,*v*_*n*−*i*−1_)), then *T*(*v*_*i*_)=_*C*_*T*(*v*_*n*−*i*+1_). Thus, *T*^*sub*^(*v*_1_,*v*_2_)≥_*C*_*T*^*sub*^(*v*_*n*_,*v*_*n*−1_).

#### **Lemma3**.

Given two different normal trees *T*_1_ and *T*_2_, *T*_1_ does not represent the same molecular graph as *T*_2_.

#### *Proof*.

We assume that *T*_1_ represents the same molecular graph as *T*_2_. Let *G*_1_ and *G*_2_ be molecular graphs transformed from *T*_1_ and *T*_2_, respectively, where each carbon in benzene rings and naphthalene rings is connected with adjacent atoms according to carbon position lists of *T*_1_ and *T*_2_. From the assumption, there is an isomorphism *ψ* from *G*_1_ to *G*_2_. It means that *l*(*v*_1_)=*l*(*ψ*(*v*_1_)) for all *v*_1_∈*V*(*G*_1_), (*ψ*(*v*_1_),*ψ*(*v*_2_))∈*E*(*G*_2_) if and only if (*v*_1_,*v*_2_)∈*E*(*G*_1_), and *mul*(*ψ*(*v*_1_),*ψ*(*v*_2_))=*mul*(*v*_1_,*v*_2_).

Consider the case that the automorphism group *Aut*(*G*_1_) of *G*_1_ has only elements *ϕ* such that *ϕ*(*v*_1_)≠*v*_2_ for *v*_1_ and *v*_2_ belonging to distinct benzene rings. Let *T*(*G*) be the molecular tree without carbon position lists, obtained from *G* by contracting benzene rings and naphthalene rings to benzene nodes, and satisfying conditions 1, 2 of Definition 6. We suppose that maps *ψ* and *ϕ* in *G*_1_ are naturally extended to *T*(*G*_1_). Since *T*_1_ is different from *T*_2_, there is a benzene node *v*_1_∈*V*(*T*_1_) such that

(3) $$\begin{array}{@{}rcl@{}} C^{T_{1}}_{v_{1}}\neq C^{T_{2}}_{\psi(v_{1})}. \end{array} $$

If *v*_1_ is not connected by a merge bond with the parent node, there is a non-identity map *ϕ*_*b*_∈*Aut*_*b*_ such that $C^{T_{1}}_{v_{1}}=\phi _{b}\left (C^{T_{2}}_{\psi (v_{1})}\right)$ because *T*_1_ and *T*_2_ represent the same molecular graph. It contradicts condition 4 of Definition 6. Suppose that *v*_1_ is connected by a merge bond with the parent node *v*_*p*_ and *Cv*_*p*_*T*_1_=*Cψ*(*v*_*p*_)*T*_2_. If $T^{sub}\left (v_{p},v_{1}\right) {=}_{\underline {C}}T^{sub}\left (v_{1},v_{p}\right)$, then *v*_*p*_ is the root, and there is a non-identity map *ϕ*_*n*_∈*Aut*_*n*_ such that $C^{T_{1}}_{(v_{p},v_{1})}=\phi _{n}\left (C^{T_{2}}_{(\psi (v_{p}),\psi (v_{1}))}\right)$ because *T*_1_ and *T*_2_ represent the same molecular graph. It contradicts condition 5a. Otherwise, $T^{sub}\left (v_{p},v_{1}\right)\neq _{\underline {C}}T^{sub}\left (v_{1},v_{p}\right)$. If *v*_*p*_ is not the root, then *T*_1_ does not represent the same molecular graph as *T*_2_ because *T*^*sub*^(*v*_*a*_,*v*_*p*_), where *v*_*a*_ is the parent of *v*_*p*_, is different from other subtrees connected to the naphthalene ring. It contradicts the assumption. If *v*_*p*_ is the root, $C^{T_{1}}_{v_{p}}=\phi_{ref}\left (C^{T_{1}}_{v_{p}}\right)$ and $C^{T_{1}}_{v_{1}}=\phi_{ref}\left (C^{T_{2}}_{\psi (v_{1})}\right)$ because *T*_1_ and *T*_2_ represent the same molecular graph. It contradicts condition 5b.

Consider the case that there is an element *ϕ*∈*Aut*(*G*_1_) such that *ϕ*(*v*_1_)=*v*_2_ for *v*_1_ and *v*_2_ belonging to distinct benzene rings. Since *T*_1_ is different from *T*_2_, there is a benzene node *v*_1_∈*V*(*T*_1_) such that

(4) $$\begin{array}{@{}rcl@{}} C^{T_{1}}_{v_{1}}\neq C^{T_{2}}_{\psi(v_{1})}. \end{array} $$

Here, we suppose that conditions 3, 4, 5 are satisfied for all benzene nodes in *T*_1_ and *T*_2_. Then, there is a path from *v*_1_ to *ϕ*(*v*_1_)=*v*_*n*_, (*v*_1_,…,*v*_*n*_), in *T*_1_. Since *T*_1_ and *T*_2_ represent the same molecular graph,

(5) $$\begin{array}{@{}rcl@{}} &&T_{1}^{sub}(v_{1},v_{2})=_{C}T_{2}^{sub}(\psi(v_{n}),\psi(v_{n-1}))~\text{and}\\ &&T_{1}^{sub}(v_{n},v_{n-1})=_{C}T_{2}^{sub}(\psi(v_{1}),\psi(v_{2})). \end{array} $$

Here, we can assume that *v*_1_ is left of *v*_*n*_ and *ψ*(*v*_1_) is left of *ψ*(*v*_*n*_) without loss of generality. Then, from Proposition 6, for paths of (*v*_1_,…,*v*_*n*_) and (*ψ*(*v*_1_),…,*ψ*(*v*_*n*_)),

(6) $$\begin{array}{@{}rcl@{}} &&T_{1}^{sub}(v_{1},v_{2})\geq_{C}T_{1}^{sub}(v_{n},v_{n-1})\,\,\text{and}\\ &&T_{2}^{sub}(\psi(v_{1}),\psi(v_{2}))\geq_{C}T_{2}^{sub}(\psi(v_{n}),\psi(v_{n-1})) \end{array} $$

because *T*_1_ and *T*_2_ are normal trees. There is no carbon position lists that satisfy Eqs. (4), (6) and (7).

Therefore, *T*_1_ does not represent the same molecular graph as *T*_2_.

## Methods

We propose an algorithm BfsBenNaphEnum for enumerating chemical compounds containing benzene rings and naphthalene rings as cyclic structures. BfsBenNaphEnum utilizes our previously developed algorithms BfsSimEnum, BfsMulEnum [18], and assigns carbon position lists.

### Modification of BfsSimEnum and BfsMulEnum

Suppose that the numbers $n_{l_{i}}$ of atoms with label *l*_*i*_ for all *l*_*i*_∈*Σ*, the numbers *n*_*b*_, *n*_*n*_ of benzene rings and naphthalene rings are given. BfsBenNaphEnum introduces a special label ‘b’ representing a benzene node to *Σ* with *b*>*l*_*i*_∈*Σ* and *val*(*b*)=6, and executes BfsSimEnum to generate all non-redundant molecular trees *T* such that $num(T, l_{i})=n_{l_{i}\phantom {\dot {i}\!}}$ for *l*_*i*_∈*Σ* except *l*_*i*_=*b*,*C* and *num*(*T*,*b*)=*n*_*b*_+2*n*_*n*_, *num*(*T*,*C*)=*n*_*C*_−6*n*_*b*_−10*n*_*n*_. At this time, all edges of enumerated trees are single because BfsSimEnum generates only simple trees. Then, we modify BfsMulEnum to assign *n*_*n*_ merge bonds to edges between benzene nodes in each tree enumerated by BfsSimEnum in addition to adding $1+\sum _{l_{i}\in \Sigma, l_{i}\neq b}num(T,{l_{i}})(val(l_{i})-2)/2$ bonds to edges between usual nodes. It should be noted that multiple bonds cannot be assigned to edges connected to benzene nodes since a carbon atom in benzene rings and naphthalene rings is connected with another adjacent atom by a single bond.

### Assignment of carbon positions for molecular trees

In this algorithm, we traverse along the tree *T* from the rightmost deepest benzene node to the root in reverse BFS order because an adjacent node list depends on carbon position lists of descendant nodes. For each benzene node *v* we found, we assign a carbon position list not to violate the conditions of normal form.

**Fig. 12:**
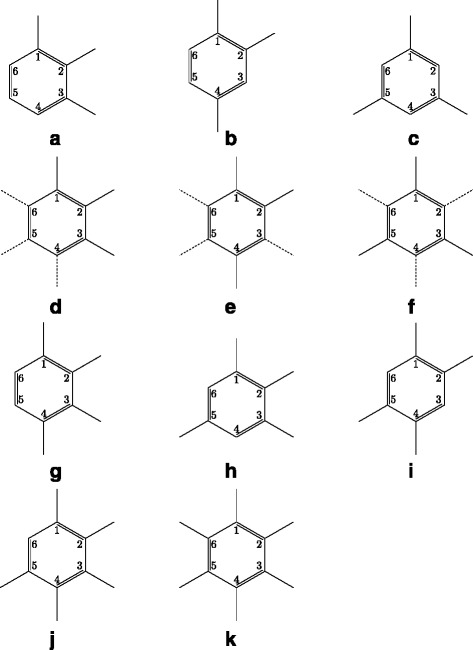
Illustration of benzene rings having each carbon position list in Table 1. **a** ((1,2,3)). **b** ((1,2,4)). **c** ((1,3,5)). **d** ((1,2,3),(4,5,6)). **e** ((1,2,4),(3,5,6)). **f** ((1,3,5),(2,4,6)). **g** ((1,2,3,4)). **h** ((1,2,3,5)). **i** ((1,2,4,5)). **j** ((1,2,3,4,5)). **k** ((1,2,3,4,5,6)). *Solid* and *dashed lines* correspond to ${A^{T}_{v}}[\!1]$ and ${A^{T}_{v}}[\!2]$, respectively

The pseudocode of assignment part in BfsBenNaphEnum is given in Algorithms 1 and 2. We always assign carbon position 1 to the first node in ${A^{T}_{v}}$ (line 20 in ASSIGN function) due to Proposition 5, which is the parent node of *v* if *v* is not the root (Proposition 2). If *v* is the root and $|{A^{T}_{v}}[\!1]|\geq 3$, we assign carbon position lists in Table 1 (see also Fig. 12) to *v* immediately for the sake of efficiency. Carbon position lists in Table 1 satisfy condition 4 of the normal form, and all the cases are included in the table.

**Table 1:** Carbon position lists for ${A^{T}_{v}}$, where *v* is the root, and $|{A^{T}_{v}}[\!1]|\geq 3$

$|{A^{T}_{v}}[\!1]|$	$|{A^{T}_{v}}[\!2]|$	${C^{T}_{v}}$
3	0	((1,2,3)), ((1,2,4)), ((1,3,5))
3	3	((1,2,3),(4,5,6)), ((1,2,4),(3,5,6)), ((1,3,5),(2,4,6))
4	0	((1,2,3,4)), ((1,2,3,5)), ((1,2,4,5))
5	0	((1,2,3,4,5))
6	0	((1,2,3,4,5,6))





For other carbon positions from 2 to 6, we use ASSIGN_CHILD to assign such positions to the remaining adjacent nodes. For example, let *T*_1_ in Fig. 6 be output without any carbon position list by BfsMulEnum. *T*_1_ has a benzene node *v*_1_, and $A^{T_{1}}_{v_{1}}=((v_{4}),(v_{5}),(v_{2},v_{3}))$. First, carbon position 1 is assigned to $A^{T_{1}}_{v_{1}}[\!1][\!1]=v_{4}$, that is, $C^{T_{1}}_{v_{1}}[\!1][\!1]=1$. Since *v*_1_ is the root and $|A^{T_{1}}_{v_{1}}[\!1]|=1<3$, Table 1 is not used, and the other nodes *v*_5_,*v*_2_,*v*_3_ are assigned by ASSIGN_CHILD. For *v*_5_, each carbon position from 2 to 6 is examined (line 26 in ASSIGN_CHILD). For *v*_2_, each position from 2 to 6 except the position assigned to *v*_5_ is examined (line 27). For *v*_3_, each position from 2 to 6 that is more than the position assigned to *v*_2_ except the position assigned to *v*_5_ is examined (line 27) because *v*_2_ and *v*_3_ have the same subtree and condition 3 must be satisfied. Thus, $C^{T_{1}}_{v_{1}}\!=((1),(2),(3,4)),((1),(2),(3,5)),((1),(2),(3,6)),\dots, ((1), (3),(2,4)),((1),(3),(2,5)),((1),(3),(2,6)),\dots,((1), (6),(4,5))$ are examined, where ((1),(6),(2,3)),((1),(6),(2,4)),((1),(5),(2,3)) and so on are discarded in the next step.

For each benzene node *v*, after assignment of a carbon position list to ${A^{T}_{v}}$, whether or not ${C^{T}_{v}}$ violates conditions 4, 5 of the normal form is confirmed (lines 5, 11, 14 in ASSIGN_CHILD). After carbon position lists are assigned to all benzene nodes, condition 6 is confirmed (line 4 in ASSIGN).

Since an input of this part, that is, an output of BfsMulEnum, satisfies conditions 1, 2 of the normal form, BfsBenNaphEnum always outputs normal trees. In ASSIGN_CHILD, a distinct carbon position list is always assigned, and all patterns are assigned (line 28). Hence, BfsBenNaphEnum outputs all distinct normal trees.

#### **Theorem1**.

BfsBenNaphEnum outputs all non-redundant molecular graphs that are solutions of Problem 1.

**Fig. 13:**
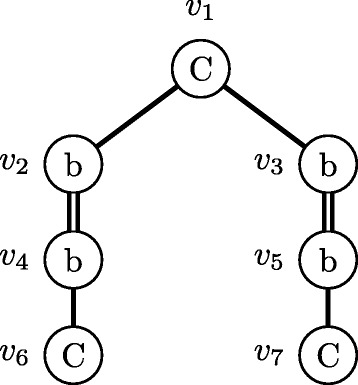
Example of a molecular tree *T*_7_

Figure 13 shows another example *T*_7_ of molecular trees. *T*_7_ includes four benzene nodes *v*_5_, *v*_4_, *v*_3_, *v*_2_ in reverse BFS order, and edges (*v*_2_,*v*_4_), (*v*_3_,*v*_5_) are merge bonds. First, our algorithm assigns carbon position lists for $A^{T_{7}}_{v_{5}}=((v_{3}),(v_{7}))$ as $C^{T_{7}}_{v_{5}}=((1,2),(3)),((1,2),(4)), ((1,2),(5)),((1,2),(6))$. In a similar way, for $A^{T_{7}}_{v_{4}}= ((v_{2}),(v_{6}))$, $C^{T_{7}}_{v_{4}}=((1,2),(3)),((1,2),(4)),((1,2),(5)), ((1,2),(6))$. For $A^{T_{7}}_{v_{3}}=((v_{1}),(v_{5}))$, $C^{T_{7}}_{v_{3}}=((1), (2,3)),((1),(3,4)),((1),(4,5)), ((1),(5,6))$ are examined. In line 5 of ASSIGN_CHILD, ((1),(4,5)) and ((1),(5,6)) are discarded because *ϕ*_*b*_(((1),(4,5)))=((1),(3,4)), *ϕ*_*b*_(((1),(5,6)))=((1),(2,3)) for the reflection map *ϕ*_*b*_ with respect to the axis through positions 1 and 4, and these violate condition 4. In a similar way, for $A^{T_{7}}_{v_{2}}=((v_{1}),(v_{4}))$, $C^{T_{7}}_{v_{2}}=((1),(2,3)),((1),(3,4))$ are assigned. After carbon position lists are assigned to all benzene nodes, condition 6 is confirmed in line 4 of ASSIGN. If *Cv*_2_*T*_7_≠*Cv*_3_*T*_7_, then there is one symmetric path, ${\mathcal {P}}=\{(v_{2},v_{3})\}$, and *T*_7_(*v*_2_)≥_*C*_*T*_7_(*v*_3_) must be satisfied. It means that *Cv*_4_*T*_7_=*Cv*_5_*T*_7_=((1,2),(3)),((1,2),(4)),((1,2),(5)),((1,2),(6)) and *Cv*_2_*T*_7_=((1),(3,4))>*Cv*_3_*T*_7_=((1),(2,3)), or *Cv*_4_*T*_7_>*Cv*_5_*T*_7_ and *Cv*_2_*T*_7_≠*Cv*_3_*T*_7_. Hence, there are $4+ {4 \choose 2}\cdot 2=16$ structures. If *Cv*_2_*T*_7_=*Cv*_3_*T*_7_=((1),(2,3)) (or *Cv*_2_*T*_7_=*Cv*_3_*T*_7_=((1),(3,4))), then ${\mathcal {P}}=\{(v_{2},v_{3}),(v_{4},v_{5})\}$, and both of *T*_7_(*v*_2_)≥_*C*_*T*_7_(*v*_3_) and *T*_7_(*v*_4_)≥_*C*_*T*_7_(*v*_5_), that is, *Cv*_4_*T*_7_≥*Cv*_5_*T*_7_, must be satisfied. Hence, there are 4+3+2+1=10 structures. In total, 16+10·2=36 structures are generated by BfsBenNaphEnum for *T*_7_.

## Results

In this section, we show that our proposed method can enumerate chemical compounds with benzene rings and naphthalene rings correctly and efficiently. For the evaluation, although MOLGEN 3.5 is more suitable than MOLGEN 5.0 to enumerate tree-like compounds because MOLGEN 3.5 offered the possibility to define substructures like benzene or naphthalene as macro atoms but MOLGEN 3.5 cannot handle all the cases provided in Table 2, we compared proposed tool with MOLGEN 5.0. Thereby, we implemented it and installed another well-known general purpose structure generator, MOLGEN 5.0, on a computer with 3.47 GHz intel Xeon CPU and 23.5 GiB memory, and compared their computational time. The implementation of BfsBenNaphEnum is available on our supplementary web site, http://sunflower.kuicr.kyoto-u.ac.jp/jira/bfsenum/.

**Table 2:** Results on execution time (sec), the number of enumerated structures by BfsBenNaphEnum and MOLGEN, and the number of chemical compounds exist in PubChem database for several instances

Chemical formula	#atoms						#all compounds in PubChem	#enumerated structures	Computational time (sec)	
	n	b	C	N	O	H			BfsBenNaphEnum	MOLGEN
*C*_7_ *O*_2_ *H*_8_	0	1	1	0	2	8	728	19	0.001	0.053
*C*_8_ *O*_3_ *H*_10_	0	1	2	0	3	10	1602	307	0.002	0.124
*C*_9_ *O*_4_ *H*_10_	0	1	3	0	4	10	1469	6406	0.010	1.699
*C*_10_ *N*_2_ *O*_4_ *H*_10_	0	1	4	2	4	10	1592	8,333,991	12.260	957.53
	1	0	0	2	4	10		7980	0.031	69.51
*C*_11_ *N*_2_ *H*_10_	0	1	5	2	0	10	790	9012	0.021	630.44
	1	0	1	2	0	10		56	0.005	24.061
*C*_12_ *N*_1_ *O*_1_ *H*_11_	0	1	6	1	1	11	1582	80,883	0.155	2,611.57
	0	2	0	1	1	11		33	0.001	98.99
	1	0	2	1	1	11		888	0.009	560.98
*C*_13_ *O*_2_ *H*_12_	0	1	7	0	2	12	1239	162,122	0.289	6,497.55
	0	2	1	0	2	12		190	0.002	2,069.3
	1	0	3	0	2	12		2458	0.013	1,731.92
*C*_14_ *O*_4_ *H*_12_	0	1	8	0	4	12	1 397	19,514,480	35.655	197,264.54
	0	2	2	0	4	12		15,581	0.021	107,509.42
	1	0	4	0	4	12		337,178	1.061	97,326.71

Since MOLGEN can enumerate chemical compounds without restriction on the structure, we must specify a benzene ring and a naphthalene ring as a substructure so that the enumerated structures contain only benzene rings and naphthalene rings as cyclic structures. As can be seen from Table 2, where ‘n’ and ‘b’ denote a naphthalene ring and a benzene ring, respectively, BfsBenNaphEnum enumerated chemical compounds much faster than MOLGEN while giving the same number of enumerated structures. BfsBenNaphEnum was from 50 times to 5,000,000 times faster than MOLGEN for instances with 8 to 14 carbon atoms. Table 2 also compares the number of discovered compounds in PubChem, which are not limited to tree-like chemical compounds, with the number of compounds enumerated by the proposed algorithm for several chemical formulas. When the number of carbon atoms is large (greater than 8 in this case), the number of discovered compounds is much less than the number of enumerated compounds. This implies that there are still a numerous number of unknown compounds to be discovered, which possibly include some essential compounds. In this study, we examined chemical formulas including up to two benzene rings and one naphthalene ring because MOLGEN was not able to output results in practical time for chemical formulas including more benzene rings and naphthalene rings.

**Fig. 14:**
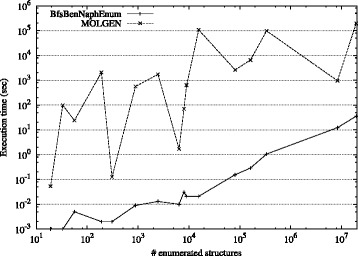
Relation between the number of enumerated structures and the computational time (sec)

We plotted the relation between the number of enumerated structures and the computational time for both methods in Fig. 14, where both x-axis and y-axis are in a log scale. It is seen from the figure that the execution time of BfsBenNaphEnum is much smaller than that of MOLGEN.

## Discussion

Our algorithm is limited to tree-like chemical structures without any cyclic structures except benzene rings and naphthalene rings while MOLGEN does not have such limitation. Therefore, in the future, we would like to extend the algorithm such that it can enumerate more complex cyclic structures, such as polycyclic aromatic compounds and nucleotides. Besides, in order to make enumeration tools practical, we need to rank enumerated structures because a large number of structures are usually enumerated. For that purpose, it might be useful to employ drug likeness filters such as Lipinski RO5, and QED score. Incorporation of such filters into our system is also important future work.

## Conclusions

We proposed a way to represent a benzene ring in a molecular tree by regarding it as a new defined atom with valence six and introducing a new attribute named carbon position list to benzene nodes. Carbon position of an atom specifies which carbon in a benzene ring that the corresponding atom bonds with. We also proposed a new kind of bond called *merge bond* that merges two benzene rings together to form a naphthalene ring. With merge bond a molecular tree can represent a structure containing naphthalene rings without defining new kind of atom. Moreover, since a benzene ring and a naphthalene ring are symmetric structures, we defined a rule to assign carbon position lists such that no redundant structures due to the symmetry of a benzene ring and a naphthalene ring are enumerated.

The algorithm of this work consists of two main steps. Given the number of benzene rings, the number of naphthalene rings as well as a chemical formula, BfsSimEnum and BfsMulEnum are applied such that they can enumerate molecular trees with benzene nodes. Next, the new extension *BfsBenNaphEnum* assigns carbon position lists to benzene nodes in normal molecular trees.

To show the performance of our algorithm, all non-redundant chemical structures were enumerated for several chemical formulas by BfsBenNaphEnum and MOLGEN 5.0, a well-known general purpose structure generator. It is shown that our algorithm is reliable since it generated the same number of structures as MOLGEN, while expended much less computational time. BfsBenNaphEnum was from 50 times to 5,000,000 times faster than MOLGEN for instances with 8 to 14 carbon atoms in our experiments. This is mainly because the number of nodes decreases from six to one for each benzene ring and from ten to two for each naphthalene ring in a chemical structure and because we enumerate chemical structures in the form of trees instead of graphs.
